# Serum creatinine as an indicator of lean body mass in vegetarians and omnivores

**DOI:** 10.3389/fnut.2022.996541

**Published:** 2022-09-16

**Authors:** Eric Bartholomae, Jessica Knurick, Carol S. Johnston

**Affiliations:** Nutrition Program, College of Health Solutions, Arizona State University, Phoenix, AZ, United States

**Keywords:** vegetarian, creatinine, lean body mass, muscle, grip strength

## Abstract

Growing numbers of Americans are adopting vegetarian or vegan diets. While risk for some chronic conditions may be lower when following these diets, concern remains over the ability to consume adequate amounts of various nutrients, notably, protein. Knowing that serum creatinine is a reliable marker of muscle mass, this study examined the relationships between serum creatinine, lean body mass (LBM), handgrip strength, and protein intake in healthy vegetarian (*n* = 55) and omnivorous (*n* = 27) adults. Significantly higher protein intakes (+31%), LBM (+7%), serum creatinine (+12%) and handgrip strength (+14%) were observed for the omnivore participants compared to vegetarian participants. Positive correlations (*p* < 0.001) were noted between creatinine and LBM (R^2^ = 0.42), creatinine and handgrip strength (R^2^ = 0.41), protein intake and LBM (R^2^ = 0.29), and handgrip strength and LBM (R^2^ = 0.69). These data show that serum creatinine concentrations were lower in vegetarian women and men in comparison to their omnivorous counterparts and that serum creatinine concentrations correlate with LBM and strength in healthy adults, regardless of diet.

## Introduction

In recent years vegetarian diets have increased in popularity. A 2020 Harris Poll reported that 4% of men and 7% of women surveyed over 18 years of age in the United States identified as vegetarians, with half of those identifying as vegan (1). While these diets are often, but not exclusively, associated with decreased risk for cancer (2–4), cardiometabolic diseases (3–5), diabetes (6), and obesity (7–9), the possibility of micronutrient inadequacies, including iron, vitamin B12, and vitamin D remain a concern. Furthermore, although energy intakes tend to be similar, protein intake is often significantly lower for vegetarians when compared to omnivores (10–14). This can be concerning as protein intakes are directly linked to muscle mass and strength (15–17). Following ingestion, protein is digested to component amino acids, which are rapidly absorbed. The increase in plasma amino acid concentrations, particularly the essential amino acid leucine, stimulates muscle protein synthesis (18, 19). In comparison to animal proteins, plant proteins contain lower amounts of leucine (20); moreover, plant protein is 10–20% less bioavailable due to antinutrient factors present in these foods (11, 21). Clinical trials have demonstrated that the rate of muscle protein synthesis is less for plant-based verse animal-based protein (22, 23). Indeed, decreased lean body mass (LBM) and reduced handgrip strength has been reported in vegetarians (24, 25). Since handgrip strength is an indicator of health-related quality of life irrespective of age and gender (26–28), it is important to consider this quality in vegetarians.

Serum creatinine, a normal waste product of muscle metabolism, has been shown to be a reliable marker of muscle mass since it is continually produced and filtered through the kidney (29, 30). However, many factors can impact serum creatinine levels, most notably declining kidney function. At the nephron, creatinine is filtered through the glomerulus and is not reabsorbed by the tubule; hence, elevations in serum creatinine can be utilized as a proxy for renal function. Since serum creatinine is generated from muscle, concentrations are impacted by age, sex, and body size. Equations have been developed using serum creatinine to estimate the glomerulus filtration rate (GFR) while controlling for these common confounding factors, and currently, the CKD–EPI Collaboration (Chronic Kidney Disease Epidemiology Collaboration) equation is considered the most accurate GFR measurement for clinical use (29), and GFR values <90 ml/min/1.73 m^2^ indicate declining kidney function. However, serum creatinine is influenced by other factors as well including medications, chronic illness, nutritional status and diet (31), all of which need to be considered when utilizing this measure.

Several investigators have reported lower serum creatinine concentrations in vegetarian populations in comparison to omnivores (32, 33). However, a comparison between serum creatinine, LBM, and strength based on diet adherence in a healthy adult population has not been reported. Therefore, the purpose of this investigation was to examine the relationship between serum creatinine, LBM, and handgrip strength in healthy, non-athlete vegetarian and omnivore women and men. We hypothesized that serum creatinine would be positively associated with LBM and handgrip strength in both genders, with omnivores exhibiting higher levels of creatinine, LBM, and handgrip strength than vegetarians.

## Subjects and methods

This study is a secondary analysis of a cross-sectional data set gathered from healthy, non-obese adults (body mass index [BMI] >18.5 and <30 kg/m^2^) to examine associations between indicators of bone health, bone mineral density, and diet adherence (vegetarian vs. omnivore) (34). Participants (27 omnivores and 55 vegetarians), aged 19–50 y, were recruited from the Phoenix metropolitan area using university list serves, connections with local vegetarian groups and farmers markets, and social media. Participants were healthy and free of chronic diseases by self-report; not taking prescription medications with the exception of oral contraceptives; not competitive athletes or training for an endurance event; and, if female, not recently pregnant. These exclusion criteria were designed to control covariables known to impact bone mineral density, but they were also relevant to this secondary analysis with a focus on lean body mass. Participants were classified as “vegetarian” if they reported never eating meat, fish, or poultry over the preceding year. “Omnivores” were classified as eating at least 3 servings of meat, fish, and/or poultry per week over the preceding year. Omnivores who reported less than 3 servings of flesh foods weekly [e.g., flexitarians or “meat-avoiders” (35)] were excluded from participation. All participants provided written consent and the study was approved by the Institutional Review Board at Arizona State University.

A 24-h diet recall was conducted by a trained nutrition professional using the multiple pass method, and diet data were analyzed using the Food Processor software (version 7.71; ESHA Research, Salem, OR, USA). Physical activity levels were estimated by validated questionnaire and reported as metabolic equivalents (METS) (36). A venous blood sample was collected following a 12-h fast, and serum was extracted for the creatinine analyses (Jaffé method, COBAS C311, Roche Diagnostics International Ltd, Switzerland). Glomerular filtration rate (GFR) was calculated using the CKD-EPI equation (29). Height was measured using a stadiometer, and body mass was recorded using a calibrated scale (model TBF-300A, Tanita Corporation, Tokyo, Japan). Dual energy X-ray absorptiometry (DEXA) (GE Lunar iDXA, Chicago, IL, USA) was used to measure LBM by a trained X-ray technician. Dominant arm handgrip strength was measured in triplicate while seated with the elbow flexed at 90 degrees and a neutral wrist position using a handheld dynamometer (Takei Scientific Instruments, Niigata-City, Japan). Three consecutive measures were taken, and the mean score was used for analyses.

### Statistical analyses

For this secondary analysis, relationships between dietary protein, serum creatinine, strength, and LBM in omnivore vs. vegetarian participants were examined by gender. Data not normally distributed based on the Kolmogorov–Smirnov test were transformed prior to analyses (LBM only). Following a significant multivariate analysis, univariate analyses were used to assess differences between means while controlling for covariates. Pearson correlations were used to identify relationship between variables, and multiple regression analyses were utilized to determine the predictive value of variables for LBM and handgrip strength. Statistical analyses were performed using SPSS version 24 (IBM, Armonk, NY, USA), and *p* ≤ 0.05 was considered significant. Data are reported as mean ± SD.

## Results

The study sample (*n* = 82) was composed of 55 vegetarians (67%) and 58 women (71%) (Table 1). Gender, BMI, energy intake, and physical activity did not vary between the diet groups; however, the vegetarian group was older on average than the omnivorous group (32.5 ± 8.8 and 27.2 ± 6.7 y respectively; *p* = 0.008). In subgroup analyses, the vegetarian men were less active than their omnivorous counterparts (41.1 ± 25.6 and 74.9 ± 49.0 METS respectively; *p* = 0.036); hence, age and physical activity were controlled in the remaining analyses.

**Table 1:** Age, body mass index, energy intake, and physical activity by diet adherence^a^.

		**OMN**	**VEG**	***P* value**
*n* (M/F)		8/19	16/39	0.267*
Age (y)		27.2 ± 6.7	32.5 ± 8.8	0.008
	Male	24.9 ± 3.7	34.8 ± 7.9	0.003
	Female	28.2 ± 7.5	31.6 ± 9.2	0.167
Body mass (kg)		66.8 ± 12.0	63.8 ± 11.0	0.054
	Male	76.4 ± 11.6	72.5 ± 11.0	0.043
	Female	62.8 ± 9.8	60.2 ± 8.9	0.205
Body mass index (kg/m^2^)		23.5 ± 3.1	22.4 ± 2.6	0.095
	Male	23.6 ± 2.4	23.3 ± 2.4	0.844
	Female	23.4 ± 3.4	22.0 ± 2.6	0.076
Energy intake (kcal)		2,153 ± 736	2,109 ± 613	0.746
	Male	2,689 ± 860	2,461 ± 518	0.433
	Female	1,901 ± 528	1,938 ± 589	0.828
METS (min/wk)		50.1 ± 39.0	38.8 ± 27.9	0.131
	Male	74.9 ± 49.0	41.1 ± 25.6	0.036
	Female	39.7 ± 29.6	37.8 ± 29.0	0.816

^a^Data are mean ± SD; p value for univariate analysis (gender controlled in total sample analysis); *indicates chi square analysis; METS, metabolic equivalents.

All variables tested were significantly impacted by diet type (Table 2). Significantly higher protein intakes (+31%), LBM (+7%), serum creatinine (+12%) and handgrip strength (+14%) were observed for the omnivore participants compared to vegetarian participants (p>0.05; Table 2). Additionally, the diet related differences for serum creatinine and handgrip strength were observed within gender groups. There were no significant relationships between serum creatinine and energy intake (r = −0.131; *p* = 0.289) or alcohol intake (r = −0.010; *p* = 0.934) among the participants. The average GFR measure was raised for the vegetarian participants relative to the omnivorous participants (+8%; *p* = 0.002), and this diet related difference was also observed within genders.

**Table 2:** Characteristics by diet adherence^a^.

		**OMN**	**VEG**	***P* value**
*n* (M/F)		8/19	16/39	0.267*
Dietary protein (g/kg)		1.48 ± 0.63	1.13 ± 0.46	0.046
	Male	1.95 ± 0.74	1.47 ± 0.67	0.466
	Female	1.26 ± 0.45	1.09 ± 0.45	0.262
Lean body mass (kg)		47.5 ± 11.2	44.2 ± 7.8	0.029
	Male	61.5 ± 7.9	54.2 ± 5.7	0.010
	Female	41.6 ± 5.6	40.1 ± 3.6	0.278
Serum creatinine (mg/dL)		0.86 ± 0.17	0.77 ± 0.12	0.002
	Male	1.04 ± 0.12	0.89 ± 0.09	0.003
	Female	0.79 ± 0.14	0.72 ± 0.09	0.034
GFR (mL/min/1.73 m)		104.2 ± 14.9	112.1 ± 11.8	0.002
	Male	104.0 ± 14.1	113.1 ± 8.5	0.002
	Female	104.3 ± 15.6	111.7 ± 13.0	0.022
Hand grip strength (kg)		29.9 ± 9.6	26.3 ± 7.7	0.005
	Male	41.8 ± 8.4	35.8 ± 4.8	0.009
	Female	24.9 ± 4.1	22.3 ± 4.6	0.047

^a^Data are mean ± SD; p value for univariate analysis (gender controlled in total sample analysis; age and physical activity controlled in all analyses); *indicates chi square analysis. Serum creatinine reference ranges: adult men, 0.7 to 1.2 mg/dL; adult women, 0.5 to 1.0 mg/dL. GFR (glomerular filtration rate) reference range: >90 mL/min.

**Figure 1:**
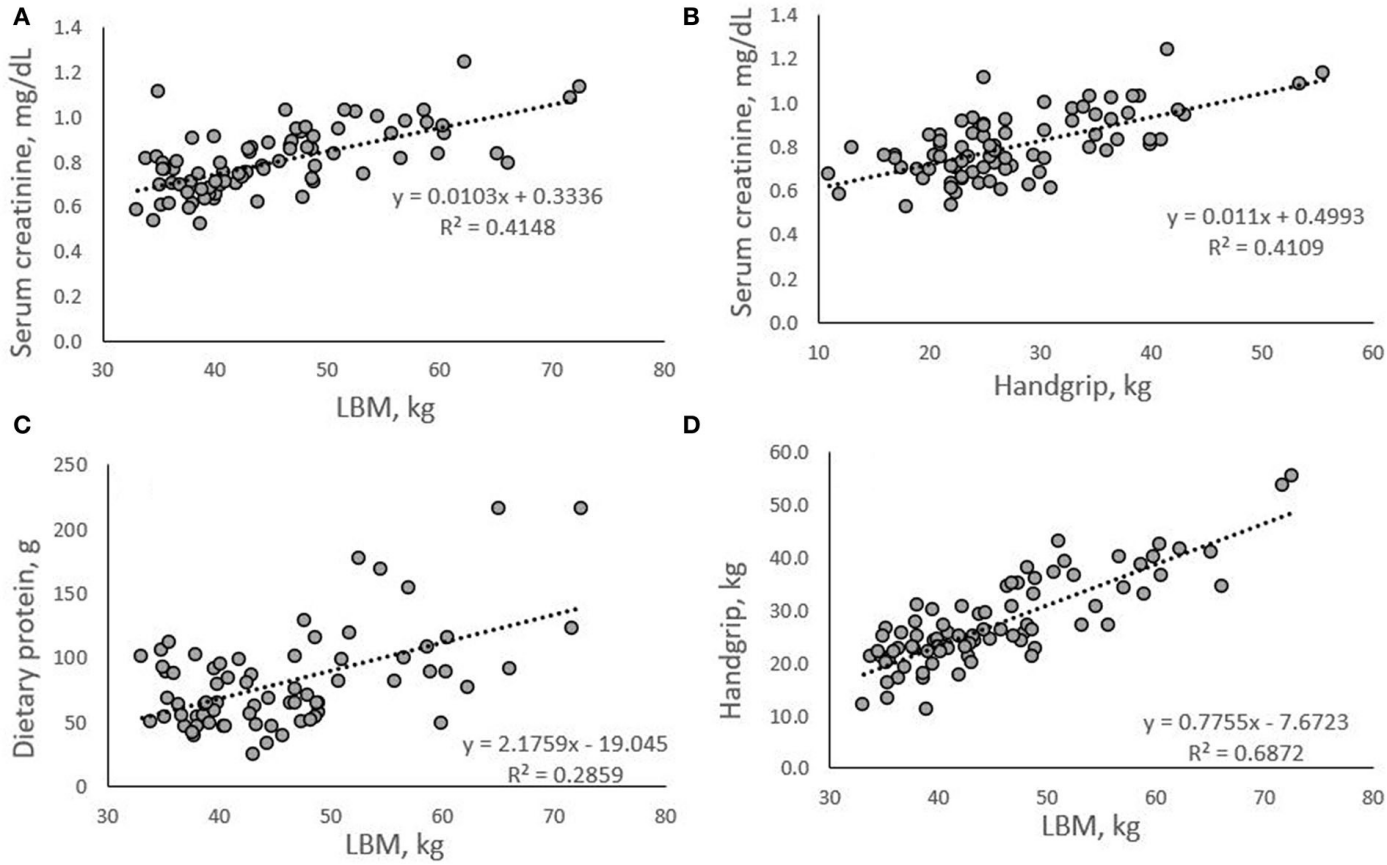
Correlations between serum creatinine, LBM **(A)**, and handgrip **(B)**; dietary protein and LBM **(C)**; and handgrip and LBM **(D)**. All correlations were significant (*p* < 0.001).

With LBM as the criterion variable, multiple regression analyses identified gender and serum creatinine as significant predictor variables (*p* < 0.05) in a model including age (*p* = 0.396), diet group (*p* = 0.316) and dietary protein (*p* = 0.111) [*F*_(5, 65)_ = 32.094, *p* < 0.001, R^2^ = 0.712] (Table 3). With handgrip strength as the criterion variable, gender, diet group, and LBM, were predictor variables (*p* < 0.05) in a model also including age (*p* = 0.614) and dietary protein (*p* = 0.423) [*F*_(5, 65)_ = 44.816, *p* < 0.001, R^2^ = 0.775] (Table 3). Correlations between serum creatinine, LBM and handgrip strength, dietary protein and LBM, and handgrip strength and LBM were significant in the study sample (*p* ≤ 0.001; Figure 1), and these significant results were retained after controlling for GFR.

**Table 3:** Multiple regression results predicting lean body mass and handgrip strength.

	**Lean body mass**					**Handgrip strength**				
	***B***	**SE**	**Beta**	***t***	***p***	***B***	**SE**	**Beta**	***t***	***p***
Gender	−12.423	1.975	−0.623	−6.290	0.000	−8.809	1.999	−0.465	−4.407	0.000
Age	0.067	0.078	0.062	0.855	0.396	−0.033	0.064	−0.032	−0.507	0.614
Diet group	−1.543	1.526	−0.079	−1.011	0.316	−2.686	1.247	−0.145	−2.154	0.035
Dietary Protein	0.034	0.021	0.138	1.615	0.111	−0.014	0.018	−0.062	−0.806	0.423
LBM						0.448	0.101	0.472	4.446	0.000
Serum creatinine	11.900	5.875	0.187	2.026	0.047					
Constant	54.766	9.862				28.655	8.634			
*R^2^*	0.712					0.775				
Adjusted *R^2^*	0.690					0.758				
	*F*_(5, 65)_ = 32.094***					*F*_(5, 65)_ = 44.816***				

***p <0.001.

## Discussion

This investigation revealed that serum creatinine is significantly reduced in vegetarian women and men compared to their meat-eating counterparts. Moreover, serum creatinine was a strong, independent predictor of LBM. The vegetarian men and women tended to consume less protein than the omnivore men and women and, importantly, protein intake and LBM were directly correlated. Moreover, vegetarians in this study had significantly lower handgrip strength than their omnivore counterparts. These findings may have important physiological relevance, as LBM and handgrip strength are related to quality of life (26–28). Moreover, Srikanthan et al. reported a negative association between muscle mass and all-cause mortality in older Americans (37). Kang et al. found a direct correlation between decreased handgrip strength and impaired mobility, increased pain and greater discomfort in both Korean men and women, with exacerbated effects as age increased, thus leading to a poorer quality of life (26). Lower levels of physical activity are associated with both a lower quality of life and with reduced handgrip strength; hence, physical activity needs to be controlled in these types of investigations (38). Herein, adults who participated in competitive sports were excluded, and the diet related differences in handgrip strength by gender remained significant controlling for age and physical activity level.

In several large cross-sectional trials, serum creatinine concentrations were significantly reduced in vegetarian populations in comparison to omnivores (32, 33). Utilizing a randomized controlled study design, Dinu et al. reported a significant reduction in serum creatinine in adults at medium-to-low risk for cardiovascular disease randomized to a hypocaloric vegetarian diet vs. a hypocaloric Mediterranean diet for 3 months (−0.04 and +0.01 mg/dL respectively, *p* < 0.001) (39). The only dietary differences between groups were for protein and fiber; however, correlation analyses showed no significant associations between these dietary changes and change in serum creatinine. Losses in total body mass was similar between groups; however, 26% more fat mass was lost in the omnivore group in comparison to the vegetarian group suggesting a greater degree of LBM loss in the latter group, which may have contributed to the significant reduction in serum creatinine noted for this group (38). In a case report, a 65-y old male with type 2 diabetes and stage 3 kidney disease, adopted a vegetarian diet, and after four months serum creatinine concentrations fell 38% from 1.6 to 1.03 mg/dL, a reduction attributed to the loss of lean body mass (40). Kochlik et al. followed serum creatinine change in adults adhering to a meatless diet for 6 days but fed 160 g cooked chicken (e.g., approximately two servings meat equating to 35 g protein) on day 4 (41). Creatinine concentrations did not fluctuate on trial days 3, 4, 5 and 6, suggesting the dependence of plasma creatinine on muscle metabolism and not meat ingestion (41). However, Mayersohn et al. reported a 52% rise in serum creatinine 2 h after consumption of the equivalent of 225 g cooked meat (42). Animal products, primarily meat, fish, and poultry, are the sole dietary source of creatine, which is partially converted to creatinine based on the cooking method (43).

Creatinine is generated at a constant rate from the spontaneous, non-enzymatic cyclization of creatine in muscle cells (30). Due to its steady production, and the fact it is freely filtered through the glomerulus, serum creatinine is used clinically as an indicator of kidney function, and high serum concentrations of creatinine (>1.4 mg/dL) are a marker of compromised kidney function (44). In fact, acute elevations in serum creatinine are considered a potent risk factor for adverse outcomes in general in the inpatient setting (43). However, less attention is directed at creatinine concentrations in the low-normal range. Low serum creatinine concentrations can indicate acute illness, severe liver disease, and the loss of muscle mass, such as in malnutrition, muscular dystrophy, or sarcopenia in older adults (45–49). Also, serum creatinine is linked to hydration status, and concentrations are lower in dehydrated states (50, 51).

In the present report, participants were young, healthy adults, screened for chronic disease, underweight and medical conditions. Thus, this report suggests that low-normal serum creatinine concentrations were linked to low LBM, reduced strength, and vegetarian diet adherence in healthy, young adults. Participants were likely well-hydrated when tested since less than 5% of the adults had serum sodium concentrations >146 mEq/L. The vegetarian men and women averaged creatinine concentrations below the mid-point of the reference ranges of 0.7–1.2 mg/dL for men and 0.5–1.0 mg/dL for women (52). In the men, serum creatinine was below 0.95 mg/dL in 81% of vegetarians vs. 13% of omnivores (*p* = 0.002). Although not statistically significant, serum creatinine was below 0.75 mg/dL in 67% of vegetarian women vs. 42% of omnivorous women (*p* = 0.094).

Previous research indicates that creatinine can be used reliably to estimate muscle mass (30, 53) and data from the present analysis support this contention (Figure 1A). Additionally, we show a positive relationship with large effect size between serum creatinine and handgrip strength, with serum creatinine explaining 41% of variance in handgrip strength (R^2^ = 0.4109; Figure 1B). It is known that dietary protein, and specifically the amino acid leucine, leads to the upregulation and activation of anabolic signaling systems responsible for muscle protein synthesis (18, 19). We have previously shown that in vegetarians and vegans who averaged less than the protein recommended dietary allowance (RDA) value of 0.8 g/kg/d, an increase in protein intake by 18 g per day significantly increased muscular strength in the absence of a training program (54). This novel finding suggested that increasing protein intake above the RDA when intake is low has beneficial effects on strength levels in vegetarians. It is well documented that inadequate protein intakes are associated with muscle protein breakdown, leading to catabolism and functional decline (55).

Some factors presented limitations in this study. Data were analyzed from a cross-sectional trial; therefore, causation cannot be determined. Additionally, this was a secondary data analysis, thus the study and data collection procedures were not initially designed for this specific outcome variable. There are many confounding variables that need to be considered as discussed, and although many of these variables were measured and/or controlled in this investigation, others were not. Gender significantly impacted the outcome variables and must be carefully considered when interpreting these data. Furthermore, flexitarians (e.g., meat-avoiders) were excluded from the study; hence, it is not known how occasional meat consumption may impact the outcome measures. This is an important consideration for a future trial. The renal function of participants was estimated using the calculated GFR; however, the gold standard measurement of renal function involves the injection of a tracer and its clearance by the kidneys (56). A single 24-h recall was used to estimate protein intakes. Research suggests that two to three 24-h records are needed for reasonable accuracy in estimating dietary protein (57). Future studies might include randomized controlled trials examining the impact of dietary protein on the outcome measures herein while addressing the limitations encountered in this report.

## Conclusion

This study shows that both men and women who follow an omnivore diet have significantly greater dietary protein intake, serum creatinine levels, and grip strength compared to vegetarian men and women. Additionally, the data show that creatinine is positively correlated to LBM and handgrip strength, and that dietary protein is positively correlated to LBM. These data offer the possibility that in healthy adults, serum creatinine may be a complementary indicator of muscle mass and strength and can be utilized by practitioners and coaches, particularly for advising vegetarian clients. However, more work is warranted regarding the relevance of serum creatinine concentrations in the low-normal range in terms of LBM and functional strength, and it is important to consider gender, altered kidney function and physical activity in these assessments.

## Data availability statement

The original contributions presented in the study are included in the article/supplementary material, further inquiries can be directed to the corresponding author.

## Ethics statement

The studies involving human participants were reviewed and approved by Institutional Review Board at Arizona State University. The patients/participants provided their written informed consent to participate in this study.

## Author contributions

EB and CJ designed the secondary research, analyzed the data, wrote the manuscript, and had primary responsibility for the final content. JK and CJ designed the original research. EB conducted the secondary research and drafted the first version of the manuscript. All authors read and approved the final manuscript and provided critical comments on the manuscript.

## Conflict of interest

The authors declare that the research was conducted in the absence of any commercial or financial relationships that could be construed as a potential conflict of interest.

## Publisher's note

All claims expressed in this article are solely those of the authors and do not necessarily represent those of their affiliated organizations, or those of the publisher, the editors and the reviewers. Any product that may be evaluated in this article, or claim that may be made by its manufacturer, is not guaranteed or endorsed by the publisher.

## References

[1] Stahler C,. How Many People Are Vegan? How Many Eat Vegan When Eating Out? Asks the Vegetarian Resource Group. (2020). Available online at: https://www.vrg.org/journal/vj2020issue4/2020_issue4_poll_results.php (accessed July 14, 2022).

[2] Tantamango-Bartley Y Jaceldo-Siegl K Fan J Fraser G. Vegetarian diets and the incidence of cancer in a low-risk population. Epidemiol Prev Biomark. (2009) 22:286–94. 10.1158/1055-9965.EPI-12-1060 23169929 PMC3565018

[3] Dinu M Abbate R Gensini GF Casini A Sofi F. Vegetarian, vegan diets and multiple health outcomes: a systematic review with meta-analysis of observational studies. Crit Rev Food Sci Nutr. (2017) 57:3640–9. 10.1080/10408398.2016.1138447 26853923

[4] Huang T Yang B Zheng J Li G Wahlqvist ML Li D. Cardiovascular disease mortality and cancer incidence in vegetarians: a meta-analysis and systematic review. Ann Nutr Metab. (2012) 60:233–40. 10.1159/000337301 22677895

[5] Kahleova H Levin S Barnard ND. Vegetarian dietary patterns and cardiovascular disease. Prog Cardiovasc Dis. (2018) 61:54–61. 10.1016/j.pcad.2018.05.002 29800598

[6] Lee Y Park K. Adherence to a vegetarian diet and diabetes risk: a systematic review and meta-analysis of observational studies. Nutrients. (2017) 9:603. 10.3390/nu9060603 28613258 PMC5490582

[7] Viguiliouk E Kendall CW Kahleová H Rahelić D Salas-Salvadó J Choo VL. Effect of vegetarian dietary patterns on cardiometabolic risk factors in diabetes: a systematic review and meta-analysis of randomized controlled trials. Clin Nutr. (2019) 38:1133–45. 10.1016/j.clnu.2018.05.032 29960809

[8] Huang RY Huang CC Hu FB Chavarro JE. Vegetarian diets and weight reduction: a meta-analysis of randomized controlled trials. J Gen Intern Med. (2016) 31:109–16. 10.1007/s11606-015-3390-7 26138004 PMC4699995

[9] Turner-McGrievy GM Barnard ND Scialli AR. A two-year randomized weight loss trial comparing a vegan diet to a more moderate low-fat diet. Obesity (Silver Spring). (2007) 15:2276–81. 10.1038/oby.2007.270 17890496

[10] Schüpbach R Wegmüller R Berguerand C Bui M Herter-Aeberli I. Micronutrient status and intake in omnivores, vegetarians and vegans in Switzerland. Eur J Nutr. (2017) 56:283–93. 10.1007/s00394-015-1079-7 26502280

[11] Kniskern MA Johnston CS. Protein dietary reference intakes may be inadequate for vegetarians if low amounts of animal protein are consumed. Nutrition. (2011) 27:727–30. 10.1016/j.nut.2010.08.024 21167687

[12] Lesser S. The 2013 FAO report on dietary protein quality evaluation in human nutrition: Recommendations and implications. Nutr Bull. (2013) 38:421–8. 10.1111/nbu.12063

[13] Phillips SM Van Loon LJC. Dietary protein for athletes: From requirements to optimum adaptation. J Sport Sci. (2011) 29:29–38. 10.1080/02640414.2011.619204 22150425

[14] Neufingerl N Eilander A. Nutrient Intake and Status in Adults Consuming Plant-Based Diets Compared to Meat-Eaters: A Systematic Review. Nutrients. (2021) 14:29. 10.3390/nu14010029 PMID 35010904 PMC8746448

[15] Gregorio L Brindisi J Kleppinger A Sullivan R Mangano KM Bihuniak JD Kenny AM Kerstetter JE Insogna KL. Adequate dietary protein is associated with better physical performance among post-menopausal women 60-90 years. J Nutr Health Aging. (2014) 18:155–60. 10.1007/s12603-013-0391-2 24522467 PMC4433492

[16] Mishra S Goldman JD Sahyoun NR Moshfegh AJ. Association between dietary protein intake and grip strength among adults aged 51 years and over: What we eat in America, national health and nutrition examination survey 2011–2014. PLoS ONE. (2018) 13:e0191368. 10.1371/journal.pone.0191368 29364939 PMC5783368

[17] Tong TYN Key TJ Sobiecki JG Bradbury KE. Anthropometric and physiologic characteristics in white and British Indian vegetarians and non-vegetarians in the UK Biobank. Am J Clin Nutr. (2018) 107:909–20. 10.1093/ajcn/nqy042 29868910 PMC5985736

[18] Stokes T Hector AJ Morton RW McGlory C Phillips SM. Recent perspectives regarding the role of dietary protein for the promotion of muscle hypertrophy with resistance exercise training. Nutrients. (2018) 10:180. 10.3390/nu10020180 29414855 PMC5852756

[19] Tipton KD Phillips SM. Dietary protein for muscle hypertrophy. Nestle Nutr Inst Workshop Ser. (2013) 76:73–84. 10.1159/000350259 23899756

[20] Dietrich S Trefflich I Ueland PM Menzel J Penczynski KJ Abraham K. Amino acid intake and plasma concentrations and their interplay with gut microbiota in vegans and omnivores in Germany. Eur J Nutr. (2022) 61:2103–14. 10.1007/s00394-021-02790-y 35034170 PMC9106628

[21] Ciuris C Lynch HM Wharton C Johnston CS. A comparison of dietary protein digestibility, based on DIAAS scoring, in vegetarian and non-vegetarian athletes. Nutrients. (2019) 11:3016. 10.3390/nu11123016 31835510 PMC6950041

[22] Wilkinson SB Tarnopolsky MA Macdonald MJ Macdonald JR Armstrong D Phillips SM. Consumption of fluid skim milk promotes greater muscle protein accretion after resistance exercise than does consumption of an isonitrogenous and isoenergetic soy-protein beverage. Am J Clin Nutr. (2007) 85:1031–40. 10.1093/ajcn/85.4.1031 17413102

[23] Hartman JW Tang JE Wilkinson SB Tarnopolsky MA Lawrence RL Fullerton AV. Consumption of fat-free fluid milk after resistance exercise promotes greater lean mass accretion than does consumption of soy or carbohydrate in young, novice, male weightlifters. Am J Clin Nutr. (2007) 86:373–81. 10.1093/ajcn/86.2.373 17684208

[24] Aubertin-Leheudre M Adlercreutz H. Relationship between animal protein intake and muscle mass index in healthy women. Br J Nutr. (2009) 102:1803–10. 10.1017/S0007114509991310 19678968

[25] Novakova K Kummer O Bouitbir J Stoffel SD Hoerler-Koerner U Bodmer M. Effect of L-carnitine supplementation on the body carnitine pool, skeletal muscle energy metabolism and physical performance in male vegetarians. Eur J Nutr. (2016) 55:207–17. 10.1007/s00394-015-0838-9 25612929

[26] Kang SY Lim J Park HS. Relationship between low handgrip strength and quality of life in Korean men and women. Qual Life Res. (2018) 27:2571–80. 10.1007/s11136-018-1920-6 29922911

[27] Marques A Gomez-Baya D Peralta M Frasquilho D Santos T Martins J. The effect of muscular strength on depression symptoms in adults: a systematic review and meta-analysis. Int J Environ Res Public Health. (2020) 17:5674. 10.3390/ijerph17165674 32781526 PMC7460504

[28] Lee MR Jung SM Bang H Kim HS Kim YB. The association between muscular strength and depression in Korean adults: a cross-sectional analysis of the sixth Korea National Health and Nutrition Examination Survey (KNHANES VI) 2014. BMC Public Health. (2018) 18:1123. 10.1186/s12889-018-6030-4 30219042 PMC6139143

[29] Cusumano AM Tzanno-Martins C Rosa-Diez GJ. The glomerular filtration rate: from the diagnosis of kidney function to a public health tool. Front Med (Lausanne). (2021) 8:769335. 10.3389/fmed.2021.769335 34926510 PMC8675900

[30] Kashani K Rosner MH Ostermann M. Creatinine: From physiology to clinical application. Eur J Intern Med. (2020) 72:9–14. 10.1016/j.ejim.2019.10.025 31708357

[31] Thongprayoon C Cheungpasitporn W Kashani K. Serum creatinine level, a surrogate of muscle mass, predicts mortality in critically ill patients. J Thorac Dis. (2016) 8:E305–11. 10.21037/jtd.2016.03.62 27162688 PMC4842835

[32] Tong TYN Perez-Cornago A Bradbury KE Key TJ. Biomarker concentrations in white and british indian vegetarians and non-vegetarians in the UK biobank. J Nutr. (2021) 151:3168–79. 10.1093/jn/nxab192 34132352 PMC8485916

[33] Xu K Cui X Wang B Tang Q Cai J Shen X. Healthy adult vegetarians have better renal function than matched omnivores: a cross-sectional study in China. BMC Nephrol. (2020) 21:268. 10.1186/s12882-020-01918-2 32652943 PMC7353802

[34] Knurick JR Johnston CS Wherry SJ Aguayo I. Comparison of correlates of bone mineral density in individuals adhering to lacto-ovo, vegan, or omnivore diets: a cross-sectional investigation. Nutrients. (2015) 7:3416-26. 10.3390/nu7053416 PMC4446759 25970147

[35] Dagevos H Voordouw J. Sustainability and meat consumption: is reduction realistic? Sustain Sci Pract Policy. (2013) 9:2. 10.1080/15487733.2013.11908115

[36] Godin G Shephard RJ. A simple method to assess exercise behavior in the community. Can J Appl Sport Sci. (1985) 10:141–6. 4053261

[37] Srikanthan P Karlamangla AS. Muscle mass index as a predictor of longevity in older adults. Am J Med. (2014) 127:547-53. 10.1016/j.amjmed.2014.02.007 PMC4035379 24561114

[38] Haider S Luger E Kapan A Titze S Lackinger C Schindler KE et al. Associations between daily physical activity, handgrip strength, muscle mass, physical performance and quality of life in prefrail and frail community-dwelling older adults. Qual Life Res. (2016) 25:3129–38. 10.1007/s11136-016-1349-8 27363692 PMC5102974

[39] Dinu M Colombini B Pagliai G Giangrandi I Cesari F Gori A. Effects of vegetarian vs. mediterranean diet on kidney function: findings from the CARDIVEG study. Eur J Clin Invest. (2021) 51:e13576. 10.1111/eci.13576 33955547 PMC8459224

[40] Campbell TM Liebman SE. Plant-based dietary approach to stage 3 chronic kidney disease with hyperphosphataemia. BMJ Case Rep. (2019) 12:e232080. 10.1136/bcr-2019-232080 31874846 PMC6936381

[41] Kochlik B Gerbracht C Grune T Weber D. The influence of dietary habits and meat consumption on plasma 3-methylhistidine-a potential marker for muscle protein turnover. Mol Nutr Food Res. (2018) 62:e1701062. 10.1002/mnfr.201701062 29573154 PMC5969234

[42] Mayersohn M Conrad KA Achari R. The influence of a cooked meat meal on creatinine plasma concentration and creatinine clearance. Br J Clin Pharmacol. (1983) 15:227–30. 10.1111/j.1365-2125.1983.tb01490.x 6849756 PMC1427867

[43] Brosnan ME Brosnan JT. The role of dietary creatine. Amino Acids. (2016) 48:1785–91. 10.1007/s00726-016-2188-1 26874700

[44] Praught ML Shlipak MG. Are small changes in serum creatinine an important risk factor? Curr Opin Nephrol Hypertens. (2005) 5:265–70. 10.1097/01.mnh.0000165894.90748.72 15821421

[45] Laszczyńska O Severo M Mascarenhas J Paiva JA Azevedo A. Serum creatinine trajectories in real-world hospitalized patients: clinical context and short-term mortality. J Investig Med. (2020) 20:870–81. 10.1136/jim-2019-001185 32179556

[46] Beben T Rifkin DE. GFR estimating equations and liver disease. Adv Chronic Kidney Dis. (2015) 15:337–42. 10.1053/j.ackd.2015.05.003 26311594 PMC4552961

[47] Singhal S Singh S Upadhyay AD Dwivedi SN Das CJ Mohta S. Serum creatinine and cystatin C-based index can be a screening biomarker for sarcopenia in older population. Eur Geriatr Med. (2019) 19:625–30. 10.1007/s41999-019-00197-x 34652732

[48] Ostermann M Kashani K Forni LG. The two sides of creatinine: both as bad as each other? J Thorac Dis. (2016) 8:E628–E630. 10.21037/jtd.2016.05.36 27501529 PMC4958791

[49] Hari P Bagga A Mahajan P Lakshmy R. Effect of malnutrition on serum creatinine and cystatin C levels. Pediatr Nephrol. (2007) 7:1757–61. 10.1007/s00467-007-0535-x 17668246

[50] Takuwa S Ito Y Ushijima K Uchida K. Serum cystatin-C values in children by age and their fluctuation during dehydration. Pediatr Int. (2002) 2:28–31. 10.1046/j.1442-200x.2002.01499.x 11982867

[51] Lešnik A Piko N Železnik D Bevc S. Dehydration of older patients in institutional care and the home environment. Res Gerontol Nurs. (2017) 17:260–6. 10.3928/19404921-20171013-03 29156066

[52] Shimada M, Dass, B, Ejaz, AA,. Assessment of elevated creatinine. BMJ Best Practice. (2020). Available online at: https://bestpractice.bmj.com/topics/en-gb/935 (accessed July 11, 2022).

[53] Kim SW Jung HW Kim CH Kim KI Chin HJ Lee H. A new equation to estimate muscle mass from creatinine and cystatin C. PLoS One. (2016) 11:e0148495. 10.1371/journal.pone.0148495 26849842 PMC4744004

[54] Bartholomae E Incollingo A Vizcaino M Wharton C Johnston CS. Mung bean protein supplement improves muscular strength in healthy, underactive vegetarian adults. Nutrients. (2019) 11:2423. 10.3390/nu11102423 31614532 PMC6836142

[55] Thalacker-Mercer AE Drummond MJ. The importance of dietary protein for muscle health in inactive, hospitalized older adults. Ann N Y Acad Sci. (2014) 1328:1–9. 10.1111/nyas.12509 25118148

[56] Warwick J Holness J. Measurement of glomerular filtration rate. Semin Nucl Med. (2022) 52:453–66. 10.1053/j.semnuclmed.2021.12.005 35063168

[57] Pereira RA Araujo MC Lopes Tde S Yokoo EM. How many 24-h recalls or food records are required to estimate usual energy and nutrient intake? Cad Saude Publica. (2010) 26:2101–11. 10.1590/s0102-311x2010001100011 21180983

